# FDA draft guidance to improve clinical trial diversity: Opportunities for pharmacoepidemiology

**DOI:** 10.1017/cts.2023.515

**Published:** 2023-05-02

**Authors:** Elizabeth S. Russell, Elodie Aubrun, Daniela C. Moga, Sandra Guedes, Wendy Camelo Castillo, Janet R. Hardy, J. Alexander Cole, Oladayo Jagun

**Affiliations:** 1 Merck & Co., Inc., Rahway, NJ, USA; 2 Novartis Pharma AG, Basel, Switzerland; 3 University of Kentucky, College of Pharmacy, Lexington, KY, USA; 4 Ferring Pharmaceuticals A/S, Copenhagen, Denmark; 5 University of Maryland School of Pharmacy, Baltimore, MD, USA; 6 Biohaven Pharmaceuticals Holding Company Ltd., New Haven, CT, USA; 7 Alexion - AstraZeneca Rare Disease, Boston, MA, USA; 8 Pfizer Inc., New York, NY, USA

**Keywords:** Trial diversity, epidemiology, real-world data

Lack of diversity in clinical trials has long been recognized, with increased awareness and actions to improve representation in recent years [1–5]. Among other initiatives, the US Food and Drug Administration (US FDA) issued a draft guidance, “Diversity Plans to Improve Enrollment of Participants from Underrepresented Racial and Ethnic Populations in Clinical Trials” on April 13, 2022, herein referred to as the Diversity Plan [6]. This draft guidance provides a framework for a Diversity Plan focused specifically on racial and ethnic characteristics. As pharmacoepidemiologists, we are highly supportive of the objectives of this new guidance. The purpose of this Perspective is to highlight methodological implementation of the Diversity Plan and opportunities for pharmacoepidemiologic studies to facilitate its implementation and expansion.

The Plan focuses on racial and ethnic diversity while encouraging inclusion of other underrepresented populations relevant to disease areas, including sex, gender identity, age, socioeconomic status, disability, pregnancy and lactation status, and comorbidities. Implementing the spirit of this Plan requires a complete characterization of the epidemiology of the disease, including its prevalence and incidence across diverse subgroups, and potential regional or community differences. Those designing and operationalizing clinical trials should proactively consider inclusion of all populations eligible to participate in medical research, and, importantly, all who may ultimately benefit and have access to the product once available on the market. Pharmacoepidemiologic principles and methods are essential to achieve representative inclusion, starting in early-stage product development (e.g. Investigational New Drug application planning). Specifically, pharmacoepidemiologists are equipped to: (1) assess whether data source(s) are available and sufficient to provide a reasonably unbiased representation of the target population(s) for the intended product indication; (2) broadly describe the determinants and burden of disease in the target population(s); (3) identify unmet patient population needs and potential barriers for clinical trial recruitment, enrollment, and participation; and (4) inform clinical trial design (e.g. assess disease burden in different subpopulations, estimate background rates of comorbidities, determine clinically relevant follow-up based on disease progression, and end point definitions). Below, we identify current challenges to implementation of Diversity Plans, and we propose solutions.

Use of available data sources within the USA often requires trade-offs in representation, generalizability, and/or individual-level detail. Data sources shown to lack representativeness of the US population with respect to diversity include commercial health insurance claims, databases from academic tertiary care centers, and geographically limited healthcare networks [7]. Data sources with detailed data on diversity may lack representation of the general population and elicit concerns regarding loss of confidentiality. Finally, population-level data sources that contain rich data on key sociodemographic variables, such as surveys for public health objectives, may lack the individual-level nuance needed for pharmacoepidemiologic studies. These limitations underscore the critical need to assess data sources early in drug development, allowing time to combine data sources or prospectively collect new data as needed. For existing data, common barriers to assessing elements of diversity include poor quality of existing measures [8], completeness of data, and inferences drawn from existing measures about intended constructs (such as using race/ethnicity as a proxy for genetic attributes or socioeconomic status [9–10]). Given the importance of these issues in advancing diversity and inclusion and thus achieving equity in clinical trials, we encourage regulators, industry, policymakers, patient groups and advocates, and other stakeholders to consider the following actions to further develop the Diversity Plan:

*Leverage robust epidemiologic methods and analyses applied to real-world data sources to inform the goals of the Diversity Plan*. A thorough understanding of the burden of disease and unmet needs should inform diversity goals in the development of therapeutics. Diversity goals add value to clinical trial participation, as emphasized in the draft guidance, but also throughout the product life cycle. It should be considered how data may be leveraged to evaluate and select disease areas of focus in discovery and early clinical development, as well as in post-marketing implementation and outreach to a diverse population. Novel approaches for data linkages or study designs may be considered when primary data collection is not feasible. Incorporating diversity in “end-to-end” drug development ultimately benefits the design and conduct of post-approval and pharmacoepidemiologic studies.

*Establish partnerships between relevant stakeholders to develop and implement a cohesive approach to the Diversity Plan*. Involving patient groups and communities early will ensure feasibility of the Diversity Plan and help address unmet needs and barriers. Furthermore, developing the Diversity Plan and refining regulatory guidance in partnership with regulatory agencies, industry, patient groups and advocates, funding bodies, and academic partners enables progress towards achieving equity goals in clinical trials.

*Expand the Diversity Plan recommendations for enrollment in clinical trials beyond race and ethnicity representation*. As pharmacoepidemiologists, we favor a more expansive approach since it allows for greater characterization of clinical trial participants and interpretation of trial outcomes beyond what race or ethnicity categories provide. Factors that lead to inequities in clinical trial participation are complex and multifaceted, and the Diversity Plan should account for the intersection of broader elements at the individual, community, and societal levels. Thus, expansion needs to be tailored to disease areas based on a thorough analysis of the epidemiology and natural history. It is noteworthy that while the FDA draft guidance primarily focuses on enrollment plans for increasing representation of racial and ethnic minorities, the US National Institutes of Health goes further by defining other elements of what constitutes diversity in clinical trials to include representation at all levels of research staff as well (in its guidance for clinical trials, and goals 6 and 7 of the National Institute of Minority and Health Disparities strategic plan 2021–2025) [2].

For pharmacoepidemiology, the Diversity Plan is a call to action to broaden our approach to diversity and equity. Pharmacoepidemiologists have the opportunity to make meaningful contributions and facilitate achievement of diversity objectives spanning the drug development life cycle and beyond. This is only the beginning – implementation of the Diversity Plan in engaging and recruiting a representative population of participants requires new approaches that may initially affect resources and timelines. Diversity in clinical trials bolsters the evidence on efficacy and safety across populations and is a further step towards advancing health equity. Strengthening collaboration among stakeholders will help reduce barriers and enrich diversity and equity in clinical research and reduce disparities and gaps in treatment ultimately providing a positive impact on public health and patient safety.
